# A Multilevel Mental Health Intervention with Latine Newcomers: The Feasibility and Mixed Methods Results of a Community-Based Participatory Pilot Study

**DOI:** 10.3390/bs16101791

**Published:** 2026-10-01

**Authors:** Meredith A. Blackwell, David T. Lardier, Julia M. Hess, Cirila Estela Vasquez Guzman, Alexandra Hernandez-Vallant, Alexis J. Handal, Kimberly Huyser, Alejandra Lemus, Janet Ramirez, Sonia Ramirez, Norma Casas, Margarita Galvis, Dulce Medina, Antonio Baca, Jessica R. Goodkind

**Affiliations:** 1 Department of Individual, Family & Community Education, University of New Mexico, Albuquerque, NM 87131, USA; blackwell@unm.edu; 2 Department of Psychiatry and Behavioral Sciences, University of New Mexico, Albuquerque, NM 87131, USA; dalardier@salud.unm.edu; 3 Department of Pediatrics, University of New Mexico, Albuquerque, NM 87131, USA; jmhess@salud.unm.edu; 4 Department of Family Medicine, Oregon Health and Science University, Portland, OR 97239, USA; vasquest@ohsu.edu; 5 Department of Psychiatry and Human Behavior, Brown University, Providence, RI 02912, USA; alexandra_hernandez-vallant@brown.edu; 6 School of Public Health, University of Michigan, Ann Arbor, MI 48109, USA; ahandal@umich.edu; 7 Department of Sociology, University of British Columbia, Vancouver, BC V6T 1Z4, Canada; kimberly.huyser@ubc.ca; 8 Department of Sociology, University of New Mexico, Albuquerque, NM 87131, USA; lemusa@unm.edu (A.L.); dulcemedina@unm.edu (D.M.); 9 School of Medicine, University of California, Los Angeles, CA 90095, USA; janetdramirez@mednet.ucla.edu; 10 New Mexico Immigrant Law Center, Albuquerque, NM 87102, USA; sramirez@nmilc.org; 11 Encuentro, Albuquerque, NM 87102, USA; norma@encuentronm.org; 12 Centro Sávila, Albuquerque, NM 87105, USA; mgalvis@centrosavila.com; 13 Albuquerque Public Schools, Albuquerque, NM 87110, USA; brandon.baca@aps.edu

**Keywords:** community-based participatory research, immigrants, intervention, Latine, mental health, mixed methods, newcomer

## Abstract

Latine immigrants in the U.S. face discrimination, a lack of access to resources, fear of deportation, family separation, and a lack of understanding of the complex factors that have led them to seek safety and survival in a new country, factors that critically impact their mental health. Academic and community partners implemented the Immigrant Well-Being Project (IWP), a mental health intervention that partners community-based organizations (CBOs) and universities to bring Latine immigrants and university students together to engage in mutual learning and social change efforts. We assessed the feasibility of the IWP model and preliminary outcomes from a pilot implementation with three cohorts of Latine immigrant adults (N = 54) between 2018 and 2021. Employing a within-group longitudinal, mixed-methods design with qualitative interviews and quantitative surveys over 12 months (pre-intervention, post-intervention, and 6-month follow-up), we examined engagement and the impact of the 6-month intervention, as well as explored feasibility/acceptability and participants’ experiences. Multilevel growth modeling revealed significant decreases in depression and anxiety symptoms and difficulty accessing resources, with dosage and cohort significantly moderating intervention effects on these variables. Some cohorts also experienced significant decreases in PTSD symptoms and increases in satisfaction with resources. Qualitative results revealed that having student and CBO support navigating services positively impacted mental health, which highlights the importance of connecting immigrants to existing community resources. Findings are discussed in the context of COVID-19 and hostile immigration policies, emphasizing the importance of addressing social–structural determinants of mental health for immigrants.

## 1. Introduction

The United States (U.S.) hosts the largest immigrant population in the world—53.3 million foreign-born residents, representing 15.4% of the total U.S. population, with Latine^1^ immigrants comprising over half of this group (Kramer & Passel, 2025). Although most people who move to the U.S. from Latin American countries are legally considered immigrants (suggesting that they are relocating by choice), there is growing recognition that the policies guiding this conceptualization are outdated and limited. First, conferring refugee status or asylum is a complex and subjective process.^2^ Second, people forcibly displaced by climate disasters, climate change, poverty induced by neoliberal colonial interference, and gang violence—have little choice, but are still categorized as immigrants (Berchin et al., 2017). Most people who leave their homes to move to a new country fall somewhere on a continuum of forcible displacement—experiencing both “push” factors that force them out and “pull” factors that attract them to a place that they believe will be safer and more viable for them (Gold, 1992; Hamlin, 2022). Thus, we use the term immigrant throughout this paper only with the understanding/caveat that most participants in this study and many other Latine immigrants in the U.S. have experienced some degree of forcible displacement.

The social, political, legal, and economic context for Latine immigrants in the U.S. plays a major role in their mental health, contributing to worsening socio-structural inequities and health disparities, including higher rates of post-traumatic stress disorder (PTSD), depression, and other forms of psychological distress, limited material resources, lingering physical ailments, and loss of meaningful social roles and support (Bojorquez, 2026). These inequities are worsened by compounding discrimination and marginalization (Elshahat et al., 2022).

In some cases, Latine immigrants experience better physical and mental health outcomes than both non-Hispanic Whites and Latine non-immigrants who were born in the U.S., described in the literature as the Hispanic Health Paradox (HHP) or the Healthy Immigrant Effect (HIE) (Alderete et al., 2000; Aldridge et al., 2018; Elshahat et al., 2022; Escobar et al., 2000; Newbold, 2006; Vega et al., 1998). However, in recent years, the HHP/HIE seems to be fading—i.e., immigrants’ mental health begins to decline as time within the U.S. increases, perhaps due to the worsening political and economic climate and increased hostility towards immigrants (Helu-Brown & Barrio, 2020), especially those arriving since 2015 compared to that of their more established counterparts (Gearing et al., 2021). Fear of deportation, experiences of discrimination, anti-immigrant rhetoric and sentiment within their communities, and resulting social isolation can collectively exacerbate chronic stress and undermine established community coping resources (Valentín-Cortés et al., 2020).

The COVID-19 pandemic further amplified the mental health burden experienced by Latine immigrants. In addition to the intensifying anti-immigrant climate, inequitable exposure, prognosis, and aid delivered during the pandemic contributed to declining mental health and increasing inequities among Latine populations (Ormiston et al., 2023). Latine populations were about 1.5 times more likely to contract COVID then non-Hispanic Whites, and nearly twice as likely to die from COVID (Hill & Artiga, 2022). These disproportionate health outcomes were compounded by financial insecurity, grief, limited access to frontline healthcare, poor physical health, and disruptions in mental health services—particularly community-based supports (Piedra et al., 2022).

### 1.1. Role of Inequities and Chronic Stressors in Immigrant Mental Health

Unequal access to medical and legal services, persistent fear of deportation or detention, disrupted social networks, limited access to social services, and historical trauma related to unsafe migration directly lead to symptoms of depression, anxiety, and stress (Becerra et al., 2020; Rios Casas et al., 2020). Contemporary research acknowledges the role chronic or daily stressors play in mental health, and suggests interventions that reduce chronic stress, such as getting documentation, access to housing, medical care, and increased income, may be more effective to reduce mental health problems than behavioral or pharmacological treatments (LeMaster et al., 2018; Miller & Rasmussen, 2010). Miller and Rasmussen (2010, 2024) specifically proposed an ecological model of psychological distress whereby both traumatic exposure and displacement-related stressors—such as poverty, unemployment, and discrimination—contributed to risk for negative mental health outcomes, with a growing body of empirical evidence supporting this model (e.g., Blackwell et al., 2024; Hou et al., 2020). Among recently resettled refugees, we found that post-migration economic stressors mediated the relationship between migration-related trauma and post-migration emotional distress and PTSD symptoms (Goodkind et al., 2021) and that resource deprivation predicted depression, anxiety, and PTSD symptoms among recently resettled refugees. However, this latter effect did not persist in the long term, suggesting the importance of ensuring initial access to resources for newcomers to prevent the development of psychological distress (Blackwell et al., 2023). Therefore, social and structural responses to migrant needs may be as important, if not more so, than interventions focusing exclusively on mental health treatment.

### 1.2. Current Study

This growing body of research demonstrating the impact of socio-structural stressors on mental health and the increasing hostile climate for Latine immigrants led us to adapt and implement a community-based resource mobilization and mutual learning intervention, first tested with refugees from Asia, Africa, and the Middle East, with Latine immigrants. This community-engaged research study involved partnership with four immigrant-focused community-based organizations and included university and community-based researchers who collaborated to design the study, adapt and implement the intervention, and assess multilevel outcomes.

We hypothesized that immigrant participants enrolled in the 6-month intervention would exhibit measurable improvements across psychological, resource-related, and quality-of-life domains over time. Outcomes were assessed longitudinally at three time points: pre- and post-intervention, and at 6-month follow-up. Specifically, we expected reductions in psychological distress, migration-related stressors, and chronic stress, as well as decreased difficulty obtaining essential resources. Concurrently, we anticipated increases in perceived social support, satisfaction with available resources, and overall quality of life across the intervention period. The current study aims to advance the science of multilevel interventions and health equity through the conceptualization of the IWP intervention model as one component of complex processes and interventions that aim to create sustainable change at multiple levels.

## 2. Materials and Methods

### 2.1. Immigrant Well-Being Project (IWP) Intervention

The IWP intervention is an adaptation of the Refugee Well-being Project (RWP) intervention, which promotes a sustainable and replicable partnership model between CBOs and universities- by bringing together newcomers and university students to: (a) enhance newcomers’ ability to navigate their communities; (b) improve access to community resources; (c) elevate meaningful social roles by valuing newcomers’ culture, experiences, and knowledge; (d) reduce social isolation among newcomers; and (e) increase communities’ responsiveness to newcomers through policy and practice changes. The intervention is administered by university students enrolled in a two-semester course and consists of two components: (1) Learning Circles, which facilitate cultural exchange and one-on-one learning opportunities, and (2) Advocacy, which focuses on collaborative efforts to mobilize community resources related to health, housing, employment, education, and legal issues. Two-hour Learning Circles occur weekly for six months (20 total). Advocacy begins after the first two Learning Circles. For the remaining 5½ months, students spend an additional 4–6 h each week with their newcomer partner(s) to engage in advocacy around newcomers’ unmet needs and goals. The intervention employs a comprehensive approach that addresses multiple needs (i.e., English proficiency, access to resources, social support, valued social roles). However, rather than emphasizing only what newcomers need to learn, the intervention focuses on mutual learning, through which newcomers both learn from and teach U.S. students. Thus, newcomers’ cultures, experiences, and knowledge are valued, while also providing them with opportunities to acquire new skills and knowledge. For a more detailed description of the intervention model, see Goodkind et al. (2020).

Multiple studies have demonstrated the feasibility, acceptability, and effectiveness of the RWP model (Goodkind et al., 2020; Goodkind et al., 2014; Lardier et al., 2026). Key adaptions made to the intervention for Latine immigrants (IWP model) included more emphasis on establishing trust and safety due to immigration status precarity and integrating the intervention more into existing programs at community partner organizations (to further build trust and to leverage the resources, strengths, and connections of long-standing immigrant CBOs).

### 2.2. Study Objectives and Design

As detailed in Goodkind et al. (2025), our community-based participatory research (CBPR) project began in 2017 and involved multiple collaborative steps. First, we conducted a qualitative assessment of the social/political/economic context for Latine immigrants in our state, their mental health, use of mental health services, and existing stressors and coping strategies. Next, community and university partners built on these findings in a series of quarterly retreats and biweekly research team meetings to adapt the IWP intervention model and integrate it into the existing programs and efforts of community partners. Finally, we employed a mixed method, within-group longitudinal design to assess feasibility, acceptability, and outcomes of the adapted intervention, the results of which we report here. The study was approved by the University of New Mexico Institutional Review Board (#22217).

### 2.3. Participant Recruitment

Sixty Latine immigrant adults (18+) were recruited in coordination with community partner organizations, with 56 enrolling in the study. Enrollment eligibility in terms of length of time in the United States was unrestricted to explore the utility of the IWP across recent and non-recent immigrants. This is important given research findings related to the impact of the Hispanic Health Paradox fading in recent years as hostility to immigrants increases. We enrolled 16 participants in Cohort 1 (November 2018–May 2019), 22 participants in Cohort 2 (November 2019–May 2020), and 18 participants in Cohort 3 (November 2020–May 2021). Exclusion criteria included presence of cognitive functioning problems, imminent suicide risk, or mental illness so severe as to impede participation in a group and that warrants immediate individual treatment.

### 2.4. Quantitative Data Collection

Each participant completed 3 surveys [pre-intervention, post-intervention (6 months), and follow-up (12 months)] in Spanish. The surveys were conducted by bilingual/bicultural interviewers using computer-assisted personal interview (CAPI). For additional information regarding interviewer training and interview procedures see Goodkind et al. (2025).

### 2.5. Quantitative Measures

Decisions about measurement inclusion and adaptations were made collaboratively. Most measures had been used with Spanish-speaking immigrants previously. For measures without an existing Spanish-language translation, we translated them using the TRAPD (Translation, Review, Adjudication, Pretesting, and Documentation) process (Walde & Völlm, 2023). See Table 1 for descriptive statistics related to primary study variables.

Depression and Anxiety Symptoms were assessed using the Hopkins Symptom Checklist (HSC-25; Derogatis et al., 1974), a self-report measure that has been validated in multiple immigrant/refugee populations (Hollifield et al., 2002). Respondents rate 25 items on a Likert-type scale from 1 (not at all) to 4 (extremely). Mean scores for items related to depression (15 items) and anxiety (10 items) are calculated to create separate subscale scores (range: 1–4), with higher scores indicating higher levels of emotional distress. Cronbach’s α at each time point ranged from 0.94–0.96.

PTSD Symptoms were measured with the PTSD Symptom Checklist–Civilian Version (PCL-C; Weathers et al., 1993), a 17-item self-reported measure of PTSD symptoms based on the criteria from the fourth edition of the Diagnostic and Statistical Manual of Mental Disorders (DSM-IV). Respondents rate their symptoms on a scale from 1 (not at all) to 5 (extremely). A total score is computed (range: 17–85), with higher scores reflecting more severe PTSD symptoms. Cronbach’s α at each time point ranged from 0.91–0.96.

Daily Stressors relevant to Latine immigrants were assessed with the Abbreviated Hispanic Stress Inventory for Immigrants (Cervantes et al., 2016), a 25-item measure of exposure to common stressors within Latine communities that includes five subscales: Occupational/Economic Stress, Parental Stress, Marital Stress, Immigration Stress, and Family and Culture Stress. Response options in the original scale version range from 1 (not at all worried/tense) to 5 (extremely worried/tense); we simplified the scale to include 4 response options in our study, based on community partner guidance. The total score is calculated by summing and averaging responses. Higher scores indicate more stress. Cronbach’s α at each time point ranged from 0.82–0.90.

Social Support was measured with the Multi-Sector Social Support Inventory Scale (MSSI; Layne et al., 2009), which evaluates perceived social support in three domains: family, ethnic community, and nonethnic community, based on past-month experiences. Each domain includes nine items related to subjective attachment and perceived support, rated from 0 (never) to 4 (almost always). The total score, ranging from 0 to 4, reflects higher levels of perceived social support. Cronbach’s α at each time point ranged from 0.88–0.92.

Access to Resources was measured in two ways. Satisfaction with Resources Scale (SR; Sullivan et al., 1992) asks respondents to rate their satisfaction with resources in 11 specific domains (e.g., education, healthcare, housing, employment). Items are rated on a 7-point scale and averaged, with domain-specific scores ranging from 1 to 7. Higher scores indicate greater satisfaction with available resources. Cronbach’s α at each time point for the SR scale ranged from 0.70–0.80. Difficulty Obtaining Resources Checklist (DARC; Sullivan & Bybee, 1999) asks participants to rate, on a 4-point scale, how difficult it has been or would be in the future to obtain resources across 12 domains. Difficulty accessing resources is computed as the mean difficulty score across all reported domains (range: 1–4). If participants are not accessing a resource, their rating reflects the perceived difficulty of accessing that resource in the future. Higher scores indicate more difficulty in accessing resources. Cronbach’s α at each time point ranged from 0.70–0.85.

Quality of Life was assessed by three items (life enjoyment, life meaningfulness, and ability to concentrate) from the Psychological Subscale of the World Health Organization Quality of Life Assessment (The Whoqol Group, 1998). These items are rated on a 5-point scale from 1 (not at all) to 5 (an extreme amount), and a subscale score is calculated with a possible range of 3.00 to 15.00. Higher scores reflect a higher perceived psychological quality of life. Cronbach’s α at each time point ranged from 0.81–0.86.

### 2.6. Quantitative Data Analytic Strategy

To assess the impact of the intervention on outcomes over time, including through the 6-month follow-up, multilevel growth modeling (Raudenbush & Bryk, 2002) was employed. This technique accounts for the hierarchical structure of the data, where repeated measurements (Level 1) are nested within individual participants (Level 2). This approach allows for modeling both individual trajectories and variability in outcomes. At Level 1, Time was included as a predictor to capture within-person changes across the follow-up period. At Level 2, a random intercept was specified to account for individual differences in baseline outcomes (Singer & Willett, 2003). The unconditional growth model was specified as:

Y*_ij_* = β_0_ + β_1_(Time*_ij_*) + u_0*j*_ + ϵ*_ij_*

In this model, Y*_ij_* represents the outcome variable for individual *j* at Time *i*, β_0_ is the intercept representing the baseline outcome, β_1_ reflects the fixed effect of Time, u_0j_ is the random intercept capturing subject-level variability, and ϵ*_ij_* represents residual error.

To assess the role of intervention engagement, the variable “Total Intervention Hours” was included as a predictor in the model. The revised model was:

Y*_ij_* = β_0_ + β_1_(Time*_ij_*) + β_2_ (Total Intervention Hours*_j_*) + β_3_ (Time*_ij_* × Total Intervention Hours*_j_*) + u_0j_ + ϵ*_ij_*

Here, β_3_ represents the extent to which the trajectory of change over time varies as a function of Total Intervention Hours.

Finally, to evaluate whether the effect of Time on outcomes varied by Cohort, a moderation model was tested. This model included both Cohort as a predictor and an interaction term between Time and Cohort:

Y*_ij_* = β_0_ + β_1_(Time*_ij_*) + β_2_(Cohort*_j_*) + β_3_(Time*_ij_* × Cohort*_j_*) + u_0j_ + ϵ*_ij_*

In this moderation model, β_3_ captures Cohort-specific differences in rates of change over time.

Model fit was assessed through nested model comparisons using likelihood ratio tests, which determined whether the inclusion of the moderators (Cohort and Total Intervention Hours) significantly improved model fit. Parameter estimation was performed using Full Information Maximum Likelihood (FIML), except when comparing models (Hox et al., 2017). In addition to model comparison, confidence intervals (CIs) were examined to provide a comprehensive interpretation of the results. Estimated marginal means were included to assess the effect of the predictors at different levels of the moderators (Cohort and Total Intervention Hours [dosage]). These estimated marginal means allowed for a more nuanced interpretation of intervention effects across different conditions.

Analyses were conducted among 42 participants, with all 126 possible surveys (3 per participant) completed. Missingness was primarily attributable to participant drop-out following baseline data collection, which is described in greater detail in the Results section. Multilevel modeling was used for longitudinal analyses and yields unbiased estimates of timepoint effects under this level of missing data. For outcome analyses, item-level missing values were handled using full information maximum likelihood (FIML) estimation.

A priori simulations were run (see Goodkind et al., 2025), based on a sample of 60 participants and accounting for 10% attrition (≈162 observations), assuming a fixed effect of β = 0.30, between-subject variance (τ^2^) = 0.40, and residual variance (σ^2^) = 0.60, which yielded an estimated power of 0.90. Additional simulations, varying effect sizes from 0.20 to 0.40 and intraclass correlations (ICCs) from 0.30 to 0.40, produced power estimates ranging from 0.801 to 0.991. These results provide evidence of the model’s sensitivity to both effect size and within-subject correlation, even accounting for attrition. For the examination of secondary Level-2 predictors (e.g., sex, age, time in the United States, intervention hours) as moderators, simulations using the same range of effect sizes and ICCs, with interaction effects ranging from 0.10 to 0.30, indicated power to detect these interactions between 0.70 and 0.80.

Post hoc simulation-based power analyses were conducted to evaluate the sensitivity of the multilevel models given the final analytic sample of 42 participants with up to three timepoints (maximum of 126 observations). Simulations assumed a random-intercept linear mixed-effects model with Time modeled as a continuous fixed effect. Effect sizes for the Time slope ranged from β = 0.20 to 0.30, and intraclass correlations (ICCs) ranged from 0.30 to 0.40, consistent with the assumptions used in the original a priori power analysis. Variance components were specified such that between-subject variance (τ^2^) equaled the ICC and residual variance (σ^2^) equaled 1 − ICC. Across 1000 simulations per condition, power to detect a significant linear Time effect (α = 0.05) ranged from 0.59 to 0.94. Specifically, power estimates were 0.59–0.63 for β = 0.20, 0.77–0.84 for β = 0.25, and 0.90–0.94 for β = 0.30, with higher power observed under greater within-subject correlation. These results indicate that, despite the smaller sample size, the analytic approach retained adequate power to detect modest to moderate longitudinal effects, particularly for effect sizes comparable to those anticipated a priori. Because this was a pilot study involving exploratory analyses, we did not adjust for testing multiple outcomes (Rothman, 1990).

### 2.7. Qualitative Data Collection

Qualitative interviews were conducted with participants in their preferred language by bilingual members of the study team, including university faculty, CBO research team members, graduate and undergraduate students. Qualitative interviews were conducted in conjunction with the quantitative surveys; all participants were invited to participate in the qualitative pre-interview; for subsequent interviews we invited a purposive sub-sample of 10 participants for each Cohort who were chosen to maximize diversity for gender, age, country of origin and CBO affiliation. Across the three Cohorts, a total of 136 qualitative interviews were conducted (53 pre-, 27 mid-intervention, 26 post-, and 30 follow-up interviews). Most interviews occurred in Spanish except for one participant in each Cohort who preferred to be interviewed in English. Interviewers received training in semi-structured interviewing techniques, including developing rapport with participants, facilitating conversations, the importance of empathy and role plays and were able to shadow an experienced interviewer. The interviews were semi-structured with an interview guide for the pre-interview that consisted of 30 questions about participants’ household composition, employment education, migration history, discrimination, mental health and well-being, social determinants of health, access to healthcare, the meaning of stress, current stressors and coping strategies, support networks both in the United States and transnationally. Subsequent interviews asked participants about changes in primary domains including work, education, children, health, as well as experiences with the intervention.

### 2.8. Qualitative Data Analysis

Interviews were recorded with the consent of participants and transcribed in the language of the interview by a professional transcription service. Data analyses were conducted by university and community members of the qualitative research team. Community research team members spoke Spanish as their native language; university team members were functionally bilingual, meaning, some were native Spanish speakers who had received most of their education in English, while others had received formal education in Spanish and English. The qualitative lead is not a native Spanish speaker, but has extensive language experience, including five years working in Latin America. The aim was to maintain these data in Spanish to enable analyses in the original language (e.g., reviewing of transcripts, coding, and analytical memos) to maintain participants’ voices and meanings and to ensure the participation of team members for whom Spanish was the dominant language. To ensure reflexive processes during data analyses, we employed a bilingual participatory approach developed by our team (see Hess et al., 2022 for a full description). These collaborative processes included discussion and memo-writing in Spanish that foregrounded the intersectional identities of research team members within the Latine immigrant community and emphasized the role of all research team members’ affective responses in data analysis, resulting in reflexive processes that recognized the impact of different positionalities of research team members and centered the perspectives of those with lived experience as immigrants.

Transcripts were imported into NVivo 12 (Lumivero, 2017). Data were analyzed using an abductive approach (combination of deductive and inductive coding that is guided by theory but allows for emergent themes) in the original Spanish by Spanish-speaking members of the qualitative team and then translated into English by qualitative team members who were bilingual in Spanish and English for the purposes of publication. Interviews were first autocoded by question, which involved a process of directing NVivo to group text according to each interview question. Next, additional thematic categories were abductively developed during collaborative retreats that included university and community research team members. Themes were entered into NVivo as the initial codes and those produced collaboratively marked as such. Four university members of the qualitative team proceeded to code transcripts and finalized the codebook for several weeks, then met weekly to re-name, merge, delete and define nodes. Data were analyzed thematically, using a combination of techniques suitable for a large, diverse coding team which balances structure and flexibility and responsiveness to the necessity of responding to current conditions. Coding was descriptive, emotions and values based (Charmaz, 2014; Miles et al., 2013). University research team members conducted coding by question domain; for example, all questions related to COVID-19 were identified and then were coded independently by two members of the qualitative team who reviewed all transcripts using the code book, to ensure no relevant data that fell under that theme was missed by one coder and also to have multiple qualitative team members with different experiences and positionalities identify emergent themes. Coding was discussed in weekly team meetings: individual coders identified new codes they added, codes were named, renamed and merged and a new database reflecting these changes was created and disseminated to team members each week. Descriptive coding facilitated team agreement. Interpretation and thematic development came in a later stage, wherein memos were created on specific themes or multiple themes analyzed together using NVivo’s querying functions. Memos were also created for each participant, analyzing their longitudinal interview data across time.

## 3. Results

### 3.1. Intervention Participation, Feasibility, Acceptability, and Fidelity

Intervention participation was assessed both as an indicator of intervention feasibility and acceptability and to use as a potential moderator in outcome analyses. Out of 20 weekly Learning Circles in the 20-week IWP intervention, participants attended an average of 8.12 (SD = 4.94), with a range of 0 to 17. Total Intervention Hours, which included Learning Circle time and time student advocates worked with immigrant participants, ranged from 10.00 to 237.68, with a mean of 90.52 (SD = 53.60), which is an average of >4.5 h of intervention per week. See Table 1 (by Cohort). These dosage levels suggest that most participants received a meaningful intervention. Variation in Learning Circle attendance and intervention hours reflects a large range of participant engagement, which should be understood within the context of newcomer participants’ busy, precarious, and stressful lives during times of heightened federal immigration enforcement (2017–2020) and the onset of the COVID-19 pandemic in March 2020. Although the IWP intervention was designed to help alleviate some of these stressors, it could not completely address structural issues that shaped participants’ particular situations.

In terms of acceptability, retention in the intervention was good. Out of 60 Latine immigrants invited by CBO partners to participate in the study, 56 (93.3%) agreed. Of these 56 who completed the baseline survey, 42 (75.0%) participated in the intervention and completed post-intervention and/or follow-up interviews. Among the 13 participants who were not retained, three moved out of the state or the U.S., three did not attend any intervention sessions or respond to attempts to contact them, and seven began the intervention but decided not to complete it nor participate in further data collection. These engagement and retention rates suggest that most participants found the IWP acceptable. In terms of feasibility, all CBO partners who participated in facilitating Learning Circles remained active throughout the study and reported that their involvement with the IWP was a favorite aspect of their work that helped them connect newcomer participants with other resources offered by their organizations (e.g., ESL classes, legal services, mental health treatment). To ensure the fidelity of intervention implementation, extensive process data was collected, including weekly progress reports, logbooks, and in-person supervision with student advocates, observations of Learning Circles and weekly Learning Circle planning and reflection meetings, and ending interviews with student–newcomer dyads to explore their experiences working together. These data indicated strong fidelity, while also underscoring the flexibility of the intervention to incorporate mobilization of varied resources and diverse learning activities, based on each participant’s goals and needs.

### 3.2. Intervention Outcomes

**Figure 1:**
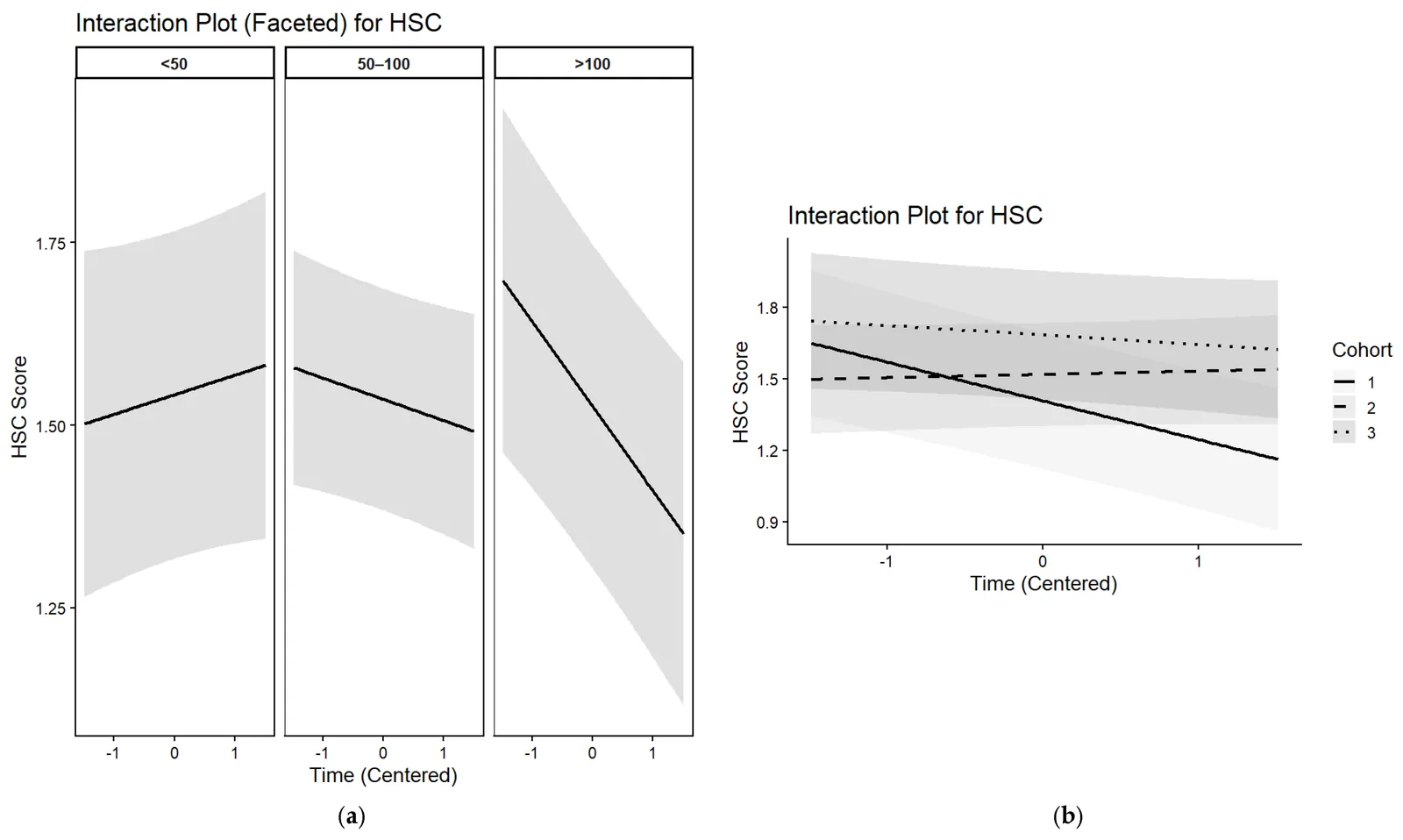
Simple slope interactions of the intervention on depression/anxiety symptoms by intervention hours (dosage) (**a**) and cohort (**b**). *Note.* HSC = Hopkins Symptom Checklist-25.

**Figure 2:**
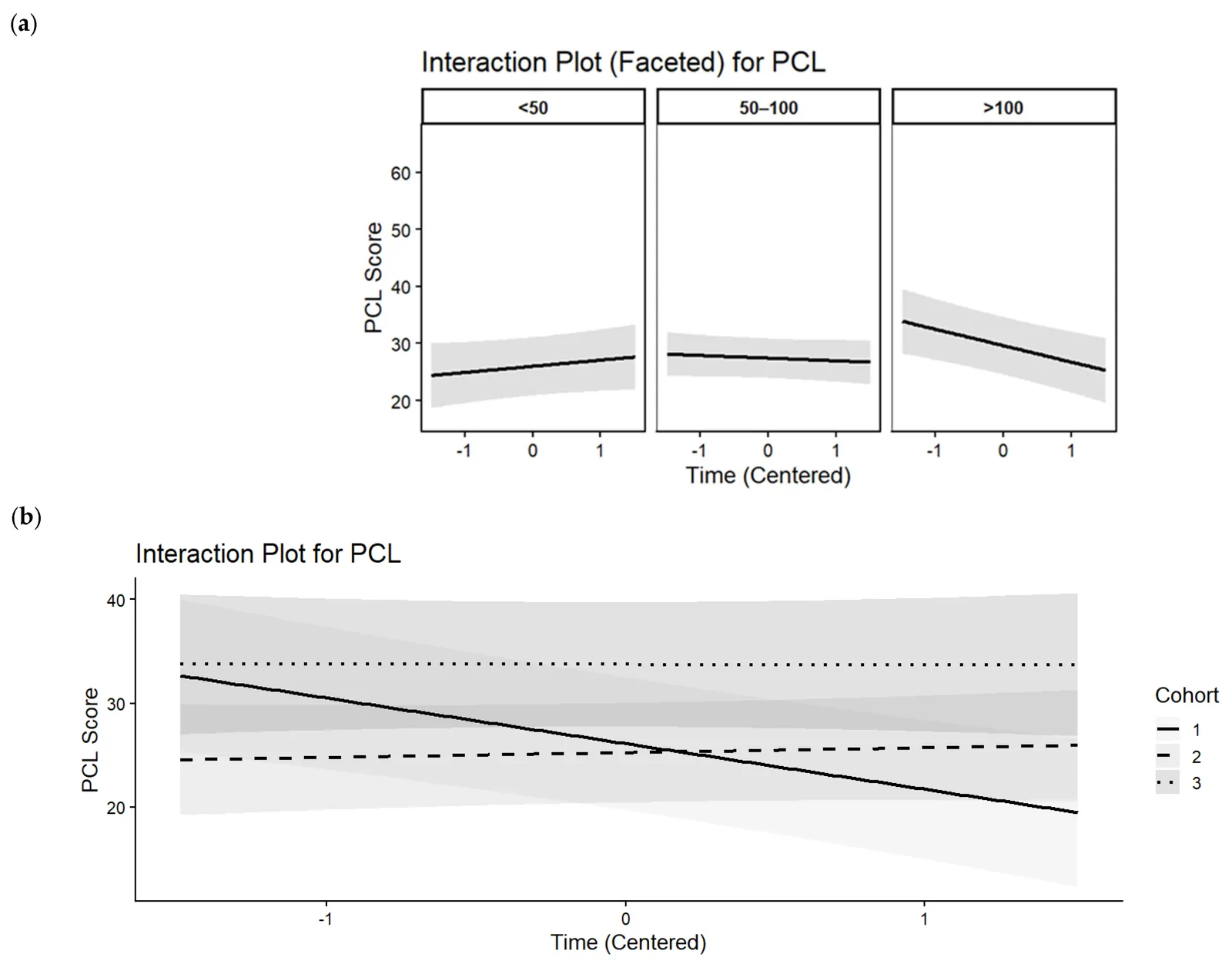
Simple slope interactions of the intervention on PTSD symptoms by intervention hours (dosage) (**a**) and cohort (**b**). *Note.* PCL = PTSD Symptom Checklist–Civilian Version.

Means, standard deviations, medians, and ranges are presented in Table 1 stratified by Cohort. Table 2 presents bivariate correlations among the study variables at baseline (pre-intervention). Table 3 presents results of mixed-effects linear modeling (MLM) on primary study outcomes. Results include MLM estimated values, standard error (SE), t-value, *p*-value and 95% CI. Figure 1 and Figure 2 contain model plots that illustrate the interaction between Time and Total Intervention Hours (dosage) and Time and Cohort on primary outcomes (see Tables S3 and S4 for estimated marginal means by dosage and cohort, respectively). The significance of interaction effects was tested using Likelihood Ratio Chi-Square tests to compare nested models. For interaction effects found to significantly improve model fit, simple slopes were computed [see Table S1 for Likelihood Ratio (LR) tests by Cohort and by dosage, respectively].

#### 3.2.1. Depression and Anxiety Symptoms

Mixed-effects linear modeling of depression/anxiety symptoms revealed a significant effect of Time (*B* = −0.04, SE = 0.01, 95% CI [−0.07, −0.01]), *p* = 0.01), demonstrating that participants’ depression/anxiety symptoms decreased significantly over time. The addition of the interaction term for Total Intervention Hours [dosage] improved model fit (LR χ^2^ [df = 2] = 12.49, *p* < 0.001). The interaction between Time and Total Intervention Hours was significant though small (Estimate = −0.001, SE = 0.0003, t = −3.59, *p* < 0.001), with greater intervention dosage/engagement associated with a steeper decline in depression/anxiety symptoms over time. Figure 1a shows the different trajectories of depression/anxiety symptoms over time for participants with low (<50 h), medium (50–100 h), and high (>100 h) intervention dosages. The mean trajectory for low-dosage participants shows an increase in depression/anxiety symptoms over time, while medium- and high-dosage participants had mean trajectories showing decreased symptoms over time, with a much steeper negative slope for high-dosage participants. The addition of the interaction term for Cohort also improved model fit (LR χ^2^ [df = 4] = 18.42, *p* < 0.001), with the interaction between Time and Cohort being significant for Cohort 2 (β = 0.17, SE = 0.04, *p* < 0.001) and Cohort 3 (β = 0.11, SE = 0.04, *p* = 0.01). These findings indicate significant variation in changes in depression/anxiety symptoms across Cohorts, with Cohorts 2 and 3 showing less decline in depression/anxiety symptoms over time compared to Cohort 1 (see Figure 1b). The mean changes in scores depicted in Figure 1 also indicate that clinically significant changes (decrease of 0.50 or greater) in depression/anxiety symptoms were achieved for participants with high intervention dosage and those in Cohort 1.

#### 3.2.2. PTSD Symptoms

Mixed-effects linear modeling of PTSD symptoms revealed a non-significant effect of Time (*p* = 0.13). However, the addition of the interaction term for Total Intervention Hours [dosage] improved model fit (LR χ^2^ [df = 2] = 10.08, *p* < 0.01). The interaction between Time and Total Intervention Hours was significant (Estimate = −0.03, SE = 0.01, t = −3.05, *p* < 0.01), indicating that a greater dosage/engagement was associated with a steeper decline in PTSD symptoms over time (see Figure 2a for mean trajectories of low-, medium-, and high-dosage participants). The addition of the interaction term for Cohort also improved model fit (LR χ^2^ [df = 4] = 17.74, *p* < 0.01), with Cohorts 2 and 3 having significant positive interactions (Estimate = 4.90, SE = 1.35, *p* < 0.001; Estimate = 4.21, SE = 1.50, *p* = 0.006 respectively). These findings indicate significant variation in changes in PTSD symptoms over time across Cohorts, with Cohorts 2 and 3 showing a slower rate of decline compared to Cohort 1 (see Figure 2b). The mean changes in scores depicted in Figure 2 suggest that clinically significant changes (decrease of 10 points or greater) in PTSD symptoms were observed for participants with high intervention dosage and those in Cohort 1.

#### 3.2.3. Difficulty Accessing Resources

Mixed-effects linear modeling for difficulty accessing resources showed marginal statistical significance (*B* = −0.11, SE = 0.06, 95% CI [−0.23, 0.001], *p* = 0.055), suggesting that participants’ difficulty accessing resources decreased significantly over time (it became easier to access resources over time). The addition of the interaction term for Total Intervention Hours [dosage] did not improve model fit (LR χ^2^ [df = 2] = 3.38, *p* = 0.18) and was not significant. Similarly, the addition of the interaction terms for Cohort did not improve model fit (LR χ^2^ [df = 4] = 3.98, *p* = 0.40) and were not significant, suggesting that decreases in difficulty accessing resources scores did not depend on intervention dosage or Cohort.

#### 3.2.4. Other Outcomes

Significant changes over time were not observed for Social Support (*p* = 0.24), Satisfaction with Resources (*p* = 0.054), Stressors (*p* = 0.051), and Quality of Life (*p* = 0.096), though some of the significance values were narrowly above the predetermined boundary of *p* < 0.05, suggesting a potentially meaningful trajectory. The addition of the interaction terms for Total Intervention Hours (dosage) and Cohort did not improve model fit for any of the variables. The interaction between Time and Total Intervention Hours was not significant; and the interaction between Time and Cohort was not significant (except for Satisfaction with Resources for Cohort 2^3^), suggesting that changes in these variables did not depend on intervention dosage or Cohort.

### 3.3. Qualitative Results

Analysis of the qualitative data revealed two interrelated outcome areas—mental health and access to resources—both of which were observed quantitatively but further elucidated through qualitative data. Participants described how their mental health and access to resources changed over the course of the IWP intervention, how these were affected by the COVID-19 pandemic (Cohorts 2 and 3), and how their immigration status shaped these experiences.

#### 3.3.1. Mental Health

Many participants reported changes in their mental health that they attributed to the intervention, which they described in a variety of ways. Participants described the Learning Circles, in particular, as leading to well-being changes: being more positive, opening up to others, and gaining inspiration or self-actualization from the experience of connecting with others. Some participants talked about the IWP intervention as stress relieving. One woman said that she was more stable, and another said that her self-esteem had increased. Participants also talked about how the experience of seeing that there are people in the community who want to help was important. Julian, a 37-year-old man from El Salvador described the change he experienced in the program this way:

Perhaps the emotional aspect is also connected to the physical one—at least, that’s how I see it—because managing stress—specifically, having a bit more emotional intelligence when it comes to handling stress—actually stimulates the body to function better. So, we learned all of that in [Learning Circles]: how to interact, how to stay united during meetings, and the experience of having others guide you—people who say, “Look, do this, do that, and if anything comes up, here’s my number—just give me a call.” That makes you feel a bit more supported. So, in that sense, it has certainly been important as well.^4^

Other intervention effects included descriptions of how the IWP student partners and staff facilitated getting urgent help for a suicidal child and helped participants access counseling, and how participants felt more social support and less alone, especially during the pandemic and/or if their immigration status was precarious. As Javier, a 31-year-old man from Mexico explained:

I realized that I’m not the only person who—who has felt afraid at times because of my immigration status; there are other people who are in an even worse situation than I am. That is what helped me a lot: seeing that I’m not the only one at this level—that there are many other people out there, and some whose circumstances are even more difficult than mine.^5^

Participants also talked about their involvement with CBOs as directly affecting their mental health. For example, Marisela, a 41-year-old woman from Peru, described how community partner organization Centro Sávila addressed her mental health:

I wasn’t resting before, it was because I couldn’t stop working. Imagine, I’m talking about not taking a month off, two months, three, four months, no, when would I stop? It was stressful, and she [Centro Sávila therapist] helped me. She also told me, “Okay, you can be here too, you can be here and we’re going to help you too, in a group so you don’t feel alone, and to complement what your therapist is helping you with.” She also helped me with things like, through organizing, paying a month’s rent, lots of things. They’ve always been there for me.^6^

Participants specifically spoke about mental health problems they and their families experienced during the pandemic and how the intervention was impactful during this time. For example, Anamaria, age 50, discussed her and her family’s history with anxiety and panic attacks and recounted how school closures due to the pandemic worsened her child’s mental health:

Yes, my daughter was very active, she was into many sports, she played several sports; in school she got into a lot of things and that kept her busy and no, she didn’t allow her anxiety to manifest itself much, I have read a lot, and whichever of those that she has [anxiety] or depression, either of which she has. Now that she is there in the house, it is really obvious, a lot of sleeping, she sleeps a lot, a lot.^7^

Idela, a Latina woman from Mexico, age 38, described how she worked hard to access care for her daughter who suffered significant mental distress during the pandemic: “When my daughter said she didn’t want to live, that same day, I sought help [from IWP staff] like you have no idea. Because, for a child, one tries everything.”^8^

Another common theme was how participants understood their mental health in relation to ongoing stressors and structural factors in their lives, including economic precarity, family separation, and stress about immigration status for themselves or loved ones, and how U.S. policies were impacting them (e.g., fear of deportation or detention), as well as policies that restricted their access to healthcare. As Agueda (age 32, from Mexico) explained:

And I don’t like that kids are listening either. Because then they start hearing “separation,” “deportation” and they start asking, and they start to worry and the kids are kids. You don’t have to worry about that. Or having that fear that their family may be separated. I don’t see why—try to avoid that for children. It’s how I think. If you inform them, in a certain way, that you have to take precautions, but don’t emphasize to them too much like, “we’re not going to go out, we’re not going to do anything,” because I feel that it’s not living. You can’t live with fear.^9^

Participants also talked about how their mental health was impacted by working conditions (e.g., economic precarity requiring work despite physical and mental health impacts; inability to work and financial impacts). For example, Alberto, a 45-year-old man from Mexico said that his greatest stressor was when work was scarce: “My main source of stress right now, as the head of the family, is when work is scarce. I get very stressed, I get in a bad mood, and I get desperate.”^10^

Discussions of these broader contextual conditions highlight the ways in which participation in the IWP supported participants’ mental health through creating a space where their current stressors were acknowledged, they were able to seek social support from peers and form new, trusting relationships with other newcomers and host community members. They learned that they had the right to ask for resources and where to access those in the community. However, the IWP data also highlight the underlying structural factors that the intervention was unable to fully mitigate or address.

#### 3.3.2. Access to Resources

Participants across all cohorts reported in post- and follow-up interviews that they had increased connections with community-based organizations (CBOs) through the IWP intervention, receiving more information about community resources and a better understanding of systems like healthcare and education. Some participants emphasized the importance of student advocates and CBOs in accessing specific resources like healthcare (e.g., signing up for the COVID-19 vaccine), assistance with communicating with their children’s schools, and accessing government benefits. Many participants mentioned how it was *only* alongside the presence of students that they were able to access the resources and services they needed. For example, Marisela, the participant from Peru, spoke about their difficulties getting an appointment to apply for unemployment:

Before I talked to them [healthcare facility] and they ignored me, right? But they [student advocates] presented themselves as students and as part of the [IWP] project and that they support me. So very quickly, they gave us a lot of information, appointments, emails, they responded and everything else.^11^

In addition to resources from students, many participants reported deepening their connections to partner CBOs through their participation in the program. CBO representatives were facilitators during Learning Circles and served several important functions. The first of which was providing participants information about existing resources in the community. For example, Dolores, a 32-year-old woman from Mexico, when asked how the program changed her life, responded:

Well, in things I didn’t know about, right? That organizations like this existed or that could help me. I didn’t know, and like I said, they helped take a weight off my shoulders. I didn’t know where to go or who to ask, and I hardly know anyone here, you know. The people I know are always working, and they don’t know about these things, but you [IWP program] do. And with someone who knows what I’m asking, you helped me. Because it’s no use asking someone else if they don’t know.^12^

In this way, CBO representatives, and the organizations themselves are fundamental sources of support for immigrants whose support networks have been strained or fractured because of migration and the stresses of post-migration life. Participants’ explanations provide support for an explicit objective of the IWP study—to adapt and embed the intervention to fit within existing efforts at CBOs that focus on mental health, education, legal services, and community mobilization for Latine immigrants.

Participants talked about the kinds of services they received from CBOs, including legal (for divorce or separation, immigration), financial support, housing, and mental health. Organizations also offered classes (e.g., English-as-a-Second Language, financial literacy). Finally, participants described how being involved in the project helped them recognize that they also enjoyed helping others. For example, Agueda, a 32-year-old woman from Mexico, said:

I discovered that I love helping others. I really enjoy helping people, I love being around children… helping the community… I took a class, I loved the topic, it’s really intense, but I like it. And I go to those information tables and it makes me so happy to be able to help people who are going through something.^13^

Thus, while participation in the IWP helped participants become connected with CBOs, which could provide them long-term resource support they might need, it also meant they could in turn provide their knowledge and expertise to others in their community through this network.

It is important to note that despite improvements in access to resources, immigration status remained a significant barrier. Many participants did not qualify to receive federal stimulus payments during the COVID pandemic and were ineligible to apply for state support. Gabriela, a 41-year-old woman from Mexico, highlighted the irony of being able to purchase a car without regular immigrant status but being denied healthcare on the same basis:

So I feel rejected by the status, because I don’t have [health] insurance, that is, I don’t have insurance because I don’t have legal status, so I say, in my country I didn’t have the opportunities, but at least I could have access to insurance or I felt safer in that aspect of being able to go to a place and anyway they took care of me.^14^

Importantly, participants recounted the role of CBOs in the community that provide essential services to immigrants, filling in gaps left by federal and state policies that systematically exclude them from services, particularly healthcare.

## 4. Discussion

This study employed a longitudinal, within-group, mixed method design to test the feasibility, acceptability, and pilot outcomes of an ecological approach to improving the mental health of Latine immigrants. The innovative intervention processes involved adapting and integrating a multilevel community-based advocacy, learning, and social support intervention (Immigrant Well-being Project, IWP) into existing efforts at four community partner organizations to address social–structural determinants of mental health through a sustainable and replicable partnership model between community-based organizations and universities. Mixed-effects linear modeling revealed significant decreases in participants’ symptoms of depression and anxiety and their difficulty accessing resources across time. Furthermore, intervention dosage significantly moderated changes in depression, anxiety, and PTSD symptoms—with higher dosage significantly related to sharper declines in mental health problems. Because this was a within-group longitudinal pilot study, our finding that intervention dosage was a significant moderator of outcomes is particularly important in supporting the premise that these changes were likely due to the IWP intervention. The large variability in intervention exposure helps with this interpretation by allowing us to examine a dose–response relationship. Because we found that intervention dosage moderated changes in mental health problems over time, our ability to make causal inferences is strengthened, and we can be more confident that the IWP intervention had meaningful positive effects on Latine immigrants’ lives. It is also important to note that intervention engagement and retention rates indicated most participants found the IWP acceptable. In terms of feasibility, all CBO partners who participated in facilitating Learning Circles remained active throughout the study and reported that their involvement with the IWP was a favorite aspect of their work. The acceptability and feasibility of the intervention was high, and effectiveness of connecting participants with CBOs was significant.

Qualitative findings underscore how the IWP intervention affected symptoms of mental health and improved resource access, as well as furthered connection with partner CBOs, who could potentially provide long-term community support services. CBOs, along with the IWP student advocates, helped participants navigate obtuse systems, such as healthcare, school, and government aid, improving their access to these critical resources, and helping them maintain stability in their lives. Our qualitative data provide powerful demonstrations of the practical impact of the intervention on participants’ lives. Although the exploratory nature of this relatively small pilot study makes it difficult to draw strong conclusions about clinically significant impact on immigrants’ mental health, the triangulation of our quantitative and qualitative findings suggest that the intervention had meaningful effects on Latine immigrants’ mental health and access to resources.

Given that many participants emphasized that being an immigrant affected their access to resources and their mental health, it is particularly important to develop, implement, and test interventions such as the IWP, which focus on building on immigrants’ strengths and addressing the unique stressors they face as newcomers to their communities who have to rebuild social support networks and navigate unknown and exclusionary systems to access needed resources (Becerra et al., 2020; LeMaster et al., 2018; Miller & Rasmussen, 2010). Our data also show the key role CBOs play for immigrants who are systematically excluded from the healthcare system, providing important access to mental, behavioral and other healthcare (Rios Casas et al., 2020).

Qualitative data also provides context for some cohort-specific differences in trajectories observed in the quantitative analyses. Reductions in depression and anxiety symptoms across time were primarily driven by larger gains made by Cohort 1, which occurred prior to the pandemic (November 2018–May 2019), while Cohort 2, which took place partially during the pandemic (November 2019–May 2020), and Cohort 3, which took place entirely during the pandemic (November 2020–May 2021), saw much smaller declines in these symptoms over time. These different contexts not only impacted outcomes, but also intervention delivery—students themselves were dealing with their own personal responses to the pandemic, and the Learning Circles were fully remote for Cohort 3 and partially remote for Cohort 2, further impacting delivery, although student advocates did have some in-person interaction with participants. For Cohort 2, it would be expected that participants’ distress could have increased (or at least not substantially decreased) from pre-pandemic to mid-pandemic. In qualitative interviews, participants reported substantial distress related to immigration status and increased difficulty accessing resources during the pandemic. It is possible that affiliation with CBOs and participation in the IWP intervention partially buffered the effect of these stressors on participants’ mental health during the pandemic. That is, these interventions may have helped prevent an otherwise larger increase in depression/anxiety symptoms during this especially stressful period, though the lack of a control group makes this impossible to say definitively. Ultimately, how and why the pandemic impacted intervention effects is speculation, but results do suggest that there was at least some effect. Given the significantly disproportionate health impact of COVID-19 on Latine populations, as well as increased mental health burden, reduction in community supports, and increased risk, a significant mental health burden among these cohorts would be expected (Hill & Artiga, 2022; Ormiston et al., 2023; Piedra et al., 2022).

Structural systems played a large role in mental health concerns and community needs. Fears over the potential for deportation and family separation, maintaining access to basic needs such as housing and paying bills, and anxiety and depression in response to pandemic-related stressors such as school closures and illness were substantial. This made the advocacy component of the IWP, which both mobilized resources to meet material needs and connected participants to CBOs, even more critical; even when the 20-weeks of Learning Circles and partnership with student advocates ended, the relationship with CBOs could continue.

Finally, qualitative results also show that connections with CBOs were seen as most beneficial when helping participants navigate structural systems and policy changes. This mechanism by which CBOs improve the mental health of immigrants by helping them navigate systems that have a direct impact on immigrant well-being was critical to elucidate. Future research needs to further investigate the role of CBOs as an effective intervention to reduce mental health problems and improve access to resources, both of which are arguably worse during periods of heightened anti-immigrant policies.

This study has several limitations. The first is the lack of a control group, which would have made it possible to definitively show intervention efficacy, rather than only demonstrating decreases in symptoms over time. As a pilot study, we sought to test the feasibility and acceptability of the intervention to see if experiences of participants justified implementing it on a broader scale. The drawback to this, however, is that it is impossible to conclude with certainty that improvements participants experienced were the result of the intervention like would be possible in a controlled trial. This concern is particularly salient given that a worldwide pandemic in the middle of the study certainly resulted in historical threats to internal validity. However, moderation analysis showing that improvements in symptoms of depression, anxiety and PTSD were moderated by intervention dosage bolsters our confidence that the intervention played a role in improvement. Moderation analysis findings related to differences across cohorts further strengthen our conclusions about intervention effects. A second weakness is the relatively small sample size; our final analytic sample was a promising start to show feasibility and acceptability for this trial but was underpowered to show more robust subgroup effects. For example, examining if participants with lower engagement differed systematically from those who received higher numbers of intervention hours would be useful to determine intervention impact, but we were unable to explore that in this sample due to its small sample size. The next step in our efforts after this pilot study is a larger randomized controlled trial that will have the power to examine subgroup effects, such as differences in effectiveness for participants of different ages, genders, and more. Finally, the confounding circumstances of the pandemic meant that not all Cohorts received the same intervention, particularly in terms of in-person vs. remote delivery.

Despite these limitations, this research demonstrates the potential effectiveness of the IWP intervention for improving the mental health of Latine immigrants. Findings indicate that a community-based intervention that collaborates with Latine immigrants, CBOs, and student advocates shows substantial promise at being acceptable and feasible, creating longstanding connections among immigrants and CBOs that can provide significant material support, and, potentially, even reducing symptoms of mental health problems and difficulty accessing resources—though future research will be necessary to verify these claims. Additionally, we find promise in the findings that this research has for the scalability and sustainability of this model in partnership with community-based organizations, which were reported as critical by participants who might not have been connected to them without this program. As recent research has found increasing emotional distress among immigrants in the wake of hostile immigration policies and discrimination (Elshahat et al., 2022), greater investment in community-level connections, intervention, and advocacy-support may help reduce distress and other mental health problems among an increasingly vulnerable population.

## Figures and Tables

**Table 1:** Sociodemographic characteristics and primary study variables by cohort at baseline.

**Categorical Variables**	**Category**	**Cohort 1 (N = 12)**	**Cohort 2 (N = 18)**	**Cohort 3 (N = 12)**
Gender	Man	3 (25.0%)	3 (16.7%)	0
	Woman	8 (66.7%)	15 (83.3%)	11 (91.7%)
	Missing	1 (8.3%)	-	1 (8.3%)
Marital Status	Single	2 (16.7%)	6 (33.3%)	5 (41.7%)
	Married	8 (66.7%)	7 (38.9%)	4 (33.3%)
	Divorced	0	2 (11.1%)	1 (8.3%)
	Widowed	1 (8.3%)	2 (11.1%)	1 (8.3%)
	Missing	1 (8.3%)	1 (5.6%)	1 (8.3%)
Country of Birth	Mexico	10 (83.3%)	16 (88.9%)	7 (58.3%)
	Other	1 (8.3%)	2 (11.1%)	4 (33.3%)
	Missing	1 (8.3%)	-	1 (8.3%)
Nationality	Mexican	11 (91.7%)	16 (88.9%)	7 (58.3%)
	Other	0	2 (11.1%)	4 (33.3%)
	Missing	1 (8.3%)	NA	1 (8.3%)
**Continuous Variables**	**Descriptives**			
Age	Mean (SD)	37.3 (10.38)	40.2 (8.58)	39.3 (9.76)
Age	Median [Range]	42.0 [18.0, 47.0]	41.0 [19.0, 51.0]	40.0 [20.0, 60.0]
Depression/Anxiety	Mean (SD)	1.42 (0.46)	1.51 (0.50)	1.65 (0.51)
Depression/Anxiety	Median [Range]	1.33 [1.00, 2.65]	1.35 [1.00, 3.48]	1.57 [1.00, 2.91]
Stressors	Mean (SD)	0.89 (0.59)	1.31 (0.68)	1.36 (0.57)
Stressors	Median [Range]	0.78 [0.00, 1.96]	1.16 [0.28, 2.74]	1.40 [0.36, 2.74]
PTSD Symptoms	Mean (SD)	26.14 (12.64)	25.20 (9.92)	33.28 (13.49)
PTSD Symptoms	Median [Range]	20.00 [17.00, 61.00]	21.00 [17.00, 66.00]	29.00 [17.00, 66.00]
Social Support	Mean (SD)	2.31 (0.62)	2.42 (0.63)	2.11 (0.93)
Social Support	Median [Range]	2.38 [0.82, 3.50]	2.54 [0.39, 3.64]	2.18 [0.39, 3.75]
Difficulty Access Res.	Mean (SD)	2.28 (0.55)	2.26 (1.12)	2.60 (0.54)
Difficulty Access Res.	Median [Range]	2.17 [1.38, 3.62]	2.00 [0.92, 9.67]	2.69 [1.50, 3.54]
Resource Satisfaction	Mean (SD)	3.28 (1.20)	3.38 (0.97)	3.22 (0.83)
Resource Satisfaction	Median [Range]	3.61 [0.50, 4.89]	3.41 [0.88, 4.88]	3.44 [1.67, 4.67]
Quality of Life	Mean (SD)	10.78 (1.85)	10.59 (2.35)	9.63 (2.68)
Quality of Life	Median [Range]	10.50 [6.00, 15.00]	11.00 [3.00, 15.00]	11.00 [5.00, 14.00]
Total Intervention Hours	Mean (SD)	123.59 (20.08)	55.37 (25.83)	118.76 (66.80)
Total Intervention Hours	Median [Range]	123.70 [87.00, 149.00]	64.50 [10.00, 90.00]	91.00 [35.76, 237.68]
Total Learning Circles	Mean (SD)	6.70 (5.78)	8.65 (4.27)	8.75 (5.33)
Total Learning Circles	Median [Range]	6.50 [0.00, 16.00]	9.00 [0.00, 15.00]	8.50 [2.00, 17.00]

*Note*. Standard Deviation = SD.

**Table 2:** Means, standard deviations, and correlations with confidence intervals (CIs) at timepoint 1 (N = 42).

Variable	1	2	3	4	5	6
1. Depress/Anx						
2. Stressors	0.25					
	[−0.05, 0.51]					
3. PTSD Symp	0.84 **	0.17				
	[0.74, 0.91]	[−0.13, 0.45]				
4. Social Support	−0.16	−0.00	−0.01			
	[−0.42, 0.12]	[−0.30, 0.29]	[−0.28, 0.27]			
5. Diff Acc Res	0.26	0.00	0.21	−0.02		
	[−0.04, 0.52]	[−0.31, 0.31]	[−0.10, 0.48]	[−0.32, 0.28]		
6. Resource Satis	−0.51 **	−0.35 *	−0.38 *	0.39*	−0.17	
	[−.072, −0.22]	[−0.62, −0.01]	[−0.63, −0.05]	[0.06, 0.64]	[−0.48, 0.17]	
7. Quality of Life	−0.58 **	−0.10	−0.39 **	0.48 **	−0.26	0.71 **
	[−.074, −0.36]	[−0.39, 0.20]	[−0.60, −0.13]	[0.24, 0.67]	[−0.52, 0.04]	[0.49, 0.84]

*Note.* Mean = M; Standard Deviation = SD. Values in square brackets indicate the 95% confidence interval for each correlation. The confidence interval is a plausible range of population correlations that could have caused the sample correlation. * Indicates *p* < 0.05. ** Indicates *p* < 0.01.

**Table 3:** Mixed-effects linear models on primary outcomes (N = 42; N observations = 126).

Outcome Variable	Effect	Estimate	SE	t-Value	*p*-Value	CI Lower	CI Upper
Depress/Anx. Symp	Time	−0.043	0.018	−2.457	0.016	−0.078	−0.009
	Intervention Hours	0.000	0.001	−0.100	0.921	−0.003	0.003
	Time:Intervention Hours	−0.001	0.000	−3.597	0.001	−0.002	−0.001
	Time:Cohort 2	0.175	0.041	4.216	0.000	0.094	0.256
	Time:Cohort 3	0.119	0.046	2.585	0.011	0.029	0.209
Stressors	Time	−0.062	0.031	−1.980	0.051	−0.124	−0.001
	Intervention Hours	−0.001	0.002	−0.601	0.552	−0.005	0.002
	Time:Intervention Hours	−0.001	0.001	−0.815	0.418	−0.002	0.001
	Cohort 2	0.510	0.216	2.355	0.024	0.086	0.934
	Cohort 3	0.569	0.238	2.388	0.022	0.102	1.036
	Time:Cohort 2	0.103	0.080	1.288	0.201	−0.054	0.261
	Time:Cohort 3	0.074	0.091	0.812	0.419	−0.105	0.253
PTSD Symp	Time	−0.853	0.567	−1.504	0.135	−1.965	0.258
	Intervention Hours	0.030	0.032	0.925	0.361	−0.034	0.094
	Time:Intervention Hours	−0.033	0.011	−3.051	0.003	−0.054	−0.012
	Cohort 2	−0.798	3.691	−0.216	0.830	−8.032	6.437
	Cohort 3	7.389	4.038	1.830	0.074	−0.525	15.304
	Time:Cohort 2	4.906	1.352	3.629	0.000	2.257	7.556
	Time:Cohort 3	4.214	1.502	2.805	0.006	1.269	7.159
Social Support	Time	−0.034	0.029	−1.179	0.241	−0.091	0.023
	Intervention Hours	−0.002	0.002	−1.035	0.308	−0.006	0.002
	Time:Intervention Hours	0.000	0.001	−0.098	0.922	−0.001	0.001
	Cohort 2	0.096	0.256	0.373	0.711	−0.407	0.598
	Cohort 3	−0.170	0.280	−0.606	0.548	−0.720	0.380
	Time:Cohort 2	0.032	0.074	0.440	0.661	−0.112	0.177
	Time:Cohort 3	−0.012	0.082	−0.141	0.888	−0.172	0.149
Diff Accessing Resources	Time	−0.116	0.060	−1.945	0.055	−0.233	0.001
	Intervention Hours	0.002	0.002	1.123	0.270	−0.002	0.006
	Time:Intervention Hours	−0.002	0.001	−1.395	0.166	−0.004	0.001
	Cohort 2	−0.042	0.234	−0.177	0.860	−0.501	0.418
	Cohort 3	0.263	0.257	1.023	0.313	−0.241	0.767
	Time:Cohort 2	0.138	0.150	0.917	0.361	−0.157	0.433
	Time:Cohort 3	−0.058	0.168	−0.348	0.728	−0.387	0.270
Resource Satis	Time	0.095	0.048	1.961	0.054	0.000	0.190
	Intervention Hours	−0.001	0.003	−0.293	0.771	−0.006	0.005
	Time:Intervention Hours	0.001	0.001	1.221	0.226	−0.001	0.003
	Cohort 2	0.301	0.363	0.831	0.411	−0.409	1.012
	Cohort 3	0.162	0.393	0.413	0.682	−0.609	0.933
	Time:Cohort 2	−0.301	0.118	−2.548	0.013	−0.533	−0.070
	Time:Cohort 3	−0.277	0.137	−2.026	0.046	−0.545	−0.009
Quality of Life	Time	−0.185	0.110	−1.681	0.096	−0.401	0.031
	Intervention Hours	−0.002	0.006	−0.329	0.744	−0.015	0.010
	Time:Intervention Hours	0.001	0.002	0.639	0.524	−0.003	0.006
	Cohort 2	−0.070	0.757	−0.093	0.926	−1.554	1.413
	Cohort 3	−1.133	0.829	−1.367	0.179	−2.758	0.491
	Time:Cohort 2	−0.275	0.277	−0.994	0.322	−0.818	0.267
	Time:Cohort 3	−0.450	0.308	−1.463	0.146	−1.053	0.153

## Data Availability

The data presented in this study are available on request from the corresponding author due to the small sample size and importance of maintaining newcomer participants’ safety.

## Notes

1 The use of the term “Latine” in this project is the result of discussion among community and university research team members and feedback from additional community members. This term was preferred over Latinx or Latino/a, as it includes a gender-neutral suffix that is found organically in the Spanish language.

2 The official definition of a refugee as defined by the UNHCR is a person who has been forcibly displaced outside of their country of nationality because of a “well-founded fear of being persecuted for reasons of race, religion, nationality, membership of a particular social group or political opinion” (UNHCR, 2007).

3 Mixed-effects linear modeling indicated a significant increase in satisfaction with resources over time (Estimate = 0.323, SE = 0.099, *p* = 0.002). In the full model, fixed-effect interactions between Time and Cohort were significant for Cohort 2 (Estimate = −0.301, SE = 0.118, *p* = 0.013) and Cohort 3 (Estimate = −0.277, SE = 0.137, *p* = 0.046), indicating attenuated increases over time relative to Cohort 1. However, likelihood ratio tests indicated that the addition of the Time × Cohort interaction did not significantly improve model fit (χ2(4) = 7.05, *p* = 0.13; Table S1). Accordingly, these moderation effects are reported descriptively and interpreted with caution.

4 Original Spanish: Quizás la parte emocional va conectada también con la parte física, pienso yo, pues porque el manejo del estrés, la inteligencia, tener un poco más de inteligencia emocional en cuanto al manejo del estrés, estimula un poco al cuerpo a trabajar mejor. Entonces pues aprendimos todo eso en esos talleres: el interactuar, el estar unidos en las reuniones, el hecho de ver de otras personas que te orientan, te dicen: “Mira, has esto, has lo otro y cualquier cosa, te doy mi número, me llamas”. Entonces se siente un poco más apoyado uno. Entonces en ese sentido sí ha sido importante también.

5 Original Spanish: Que me fijé que no soy la única persona que… que he tenido en veces miedo, por como estoy en migración, y hay otras personas que están peor que a uno. Eso es de lo que me ayudó mucho, mirar que no soy el único que está en ese nivel, hay muchas más personas, y hay personas que están más difíciles que uno.

6 Original Spanish: Dejé de trabajar, no percibo que me vienen todo, mucho, si no descansaba antes, era porque no podía dejar de trabajar, imagínate, lo digo sin dejar ya un mes dos, meses, tres, cuatro, no, cuándo ya, no, fue estresante y pues ella me ayudó, y me dijo también: “Okay, también puedes estar aquí, puedes estar aquí y te vamos a ayudar también, pues en un grupo para que no te sientas sola, y complementes lo que te está ayudando tu terapeuta”. Me ayudó también para, o sea, por medio de la organización para pagar un mes de renta, bastantes cosas, para qué, siempre han estado ahí.

7 Original Spanish: Sí, mi hija la chica era muy activa, ella estaba en muchos deportes, jugaba varios deportes; la escuela, se metía a muchas cosas y eso la mantenía ocupada y no permitía que su ansiedad se manifestara mucho, leyera mucho o la depresión, cualquiera de las dos tiene. Ya ahora que, que está ahí en la casa se le manifiesta con mucho, mucho dormir, duerme mucho, mucho.

8 Original Spanish: Cuando mi hija dijo que no quería vivir, yo ese mismo día, busqué ayuda como no tienes idea. Porque uno por un hijo mueve todo.

9 Original Spanish: Y no me gusta que los niños estén escuchando tampoco. Porque luego empiezan a escuchar “separación”, “deportación” y ellos empiezan a preguntar, y se empiezan a preocupar y los niños son niños. No tienen por qué preocuparse por eso. O tener ese temor de que su familia se puede separar. No miro por qué–tratar de evitarles eso a los niños. Es como yo pienso. Si informarlos, de cierta forma, que uno tiene que tener precauciones, pero no recalcarles mucho como, “no vamos a salir, no vamos a hacer nada”, porque siento que no es vivir. Con miedo no se puede vivir.

10 Original Spanish: Mi estrés principal como cabeza de familia ahorita, es cuando el trabajo está escaso. Me estreso mucho, me pongo de mal humor, me desespero.

11 Original Spanish: Como cuando antes uno le hablaba y no hacían caso, ¿no? Pero ellas se presentaron como estudiantes y como parte del proyecto y que me apoyan, ¿no? Entonces ahí sí, muy rápido, nos dieron mucha información, citas, correos, respondían y todo lo demás.

12 Original Spanish: P: En que pues en cosas que no sabía yo ¿verdad? que habían organizaciones así o que me pudieran ayudar, yo no sabía y como le digo, me ayudaron a quitarme un peso de encima, no sabía a dónde acudir o a quién preguntarle algo y como que no tengo a nadie casi aquí, pues usted sabe y las personas que conozco siempre están trabajando y pues ellos no saben de los temas y pues ustedes sí y pues con alguien que sepa de lo que me ayudaron en personas que sí saben lo que yo le estoy preguntando, porque igual de nada sirve que le pregunte a otra persona si no sabe.

13 Original Spanish: Y descubrí que me encanta ayudar. Me gusta mucho ayudar me gusta estar rodeada de niños… ayudar a la comunidad… Tomé una clase, me encantó el tema, está fuertísimo, pero me gusta. Y voy a esas mesas informativas y me dan mucha ilusión poder ayudar a personas que estén pasando por algo.

14 Original Spanish: Entonces me siento rechazada por el estatus, porque no tengo aseguranza, o sea, no tengo aseguranza porque no tengo un estatus legal entonces digo, pues en mi país no tenía las oportunidades, pero por lo menos pues sí podía tener acceso a un seguro o me sentía más segura en ese aspecto de poder ir a un lugar y pues como sea me atendían.

## Supplementary Materials

The following supporting information can be downloaded at: https://www.mdpi.com/article/10.3390/bs16101791/s1, Table S1: Log-likelihood ratio (LR) test by cohort; Table S2: Log-likelihood ratio (LR) test by dosage. Table S3: Estimated marginal means by dosage exposure. Table S4: Estimated marginal means by cohort.
