# Supplementary material for: A Multilevel Mental Health Intervention with Latine Newcomers: The Feasibility and Mixed Methods Results of a Community-Based Participatory Pilot Study
Source: Behav Sci (Basel). 2026 Oct 1;16(10):1791. doi: 10.3390/bs16101791 (PMC13701686; doi:10.3390/bs16101791)
Supplement: Supplementary file 1 [file behavsci-16-01791-s001.zip › behavsci-4381132-supplementary.pdf]

Supplementary Materials

Table S1.
Log-likelihood ratio (LR) test by cohort

Variable	LR without moderator	LR with moderator	Chi-square	df	p-value
HSC	-40.945	-31.556	18.778	4	0.000
HSI	-70.934	-68.936	3.997	4	0.406
PCL	-506.909	-498.851	16.115	4	0.002
MSSI	-109.438	-108.014	2.849	4	0.583
DARC	-146.019	-143.309	5.420	4	0.246
SR	-102.691	-99.4435	6.494	4	0.165
WHO	-286.476	-283.983	4.984	4	0.288

Note. Log Likelihood ratio = LR; Degrees of Freedom = df; Hopkins Symptom Checklist = HSC; Hispanic Stress Inventory = HSI; PTSD Symptom Checklist–Civilian Version = PCL; Multi-Sector Social Support Inventory = MSSI; Difficulty Accessing Resources Checklist = DARC; Satisfaction with Resources = SR; World Health Organization Quality of Life Scale = WHOQOL

Table S2.
Log-likelihood ratio (LR) test by dosage

Variable	LR without moderator	LR with moderator	Chi-square	df	p-value
HSC	-41.091	-33.228	15.72	2	0.000
HSI	-70.934	-69.195	3.478	2	0.175
PCL	-486.887	-480.775	12.223	2	0.002
MSSI	-106.03	-104.639	2.783	2	0.248
DARC	-144.302	-142.627	3.348	2	0.187
SR	-96.4212	-95.2883	2.265	2	0.322
WHO	-275.303	-274.979	0.646	2	0.723

Note. Hopkins Symptom Checklist = HSC; Hispanic Stress Inventory = HSI; PTSD Symptom Checklist–Civilian Version = PCL; Multi-Sector Social Support Inventory = MSSI; Difficulty Accessing Resources Checklist = DARC; Satisfaction with Resources = SR; World Health Organization Quality of Life Scale = WHOQOL

Table S3.
Estimated marginal means by dosage

**Variable**	**Exposure Level**	**Emmean**	**SE**	**df**	**Lower CL**	**Upper CL**
HSC	Low exposure (−1 SD)	1.534	0.075	35.945	1.382	1.686
HSC	Mean exposure	1.534	0.075	35.951	1.382	1.686
HSC	High exposure (+1 SD)	1.534	0.075	35.955	1.382	1.686
HSI	Low exposure (−1 SD)	1.221	0.098	33.843	1.021	1.420
HSI	Mean exposure	1.220	0.098	33.853	1.020	1.419
HSI	High exposure (+1 SD)	1.219	0.098	33.863	1.019	1.418
PCL	Low exposure (−1 SD)	27.771	1.706	35.862	24.310	31.232
PCL	Mean exposure	27.801	1.705	35.871	24.342	31.261
PCL	High exposure (+1 SD)	27.832	1.705	35.880	24.373	31.291

MSSI	Low exposure (−1 SD)	2.307	0.107	35.908	2.090	2.524
MSSI	Mean exposure	2.305	0.107	35.915	2.088	2.521
MSSI	High exposure (+1 SD)	2.303	0.107	35.922	2.086	2.519
DARC	Low exposure (−1 SD)	2.371	0.099	32.932	2.169	2.574
DARC	Mean exposure	2.373	0.099	32.940	2.171	2.576
DARC	High exposure (+1 SD)	2.375	0.099	32.947	2.173	2.578
SR	Low exposure (−1 SD)	3.344	0.151	33.617	3.038	3.650
SR	Mean exposure	3.343	0.150	33.655	3.037	3.648
SR	High exposure (+1 SD)	3.342	0.150	33.695	3.037	3.647
WHOQOL	Low exposure (−1 SD)	10.269	0.336	35.926	9.587	10.951
WHOQOL	Mean exposure	10.267	0.336	35.935	9.585	10.948
WHOQOL	High exposure (+1 SD)	10.265	0.336	35.944	9.583	10.946

Note. Estimated Marginal Means = Emmean; Confidence Level = CL; Standard Error = SE; Degrees of Freedom = df; Hopkins Symptom Checklist = HSC; Hispanic Stress Inventory = HSI; PTSD Symptom Checklist–Civilian Version = PCL; Multi-Sector Social Support Inventory = MSSI; Difficulty Accessing Resources Checklist = DARC; Satisfaction with Resources = SR; World Health Organization Quality of Life Scale = WHOQOL

Table S4.

Estimated marginal means by cohort

Measure	Cohort	Emmean	SE	df	Lower CL	Upper CL
HSC	1	1.428	0.131	40.824	1.401	1.922
HSC	2	1.503	0.105	38.167	1.291	1.716
HSC	3	1.661	0.129	38.505	1.164	1.693
HSI	1	0.804	0.172	39.721	0.457	1.152
HSI	2	1.314	0.132	34.056	1.046	1.582
HSI	3	1.373	0.165	35.155	1.038	1.708
PCL	1	25.965	2.903	42.124	20.106	31.824
PCL	2	25.167	2.282	37.371	20.544	29.790
PCL	3	33.354	2.809	38.156	27.668	39.040
MSSI	1	2.302	0.201	41.285	1.897	2.707
MSSI	2	2.398	0.160	37.915	2.074	2.721
MSSI	3	2.132	0.196	38.381	1.735	2.529
DARC	1	2.308	0.189	39.752	1.927	2.690
DARC	2	2.267	0.140	32.091	1.981	2.552
DARC	3	2.571	0.176	36.243	2.215	2.927
SR	1	3.103	0.284	37.239	2.528	3.677
SR	2	3.404	0.226	35.806	2.945	3.863
SR	3	3.265	0.273	37.463	2.712	3.818
WHOQOL	1	10.681	0.594	41.753	9.482	11.879
WHOQOL	2	10.610	0.470	37.579	9.658	11.562
WHOQOL	3	9.547	0.579	38.355	8.376	10.718

Note. Estimated Marginal Means = Emmean; Confidence Level = CL; Standard Error = SE; Degrees of Freedom = df; Hopkins Symptom Checklist = HSC; Hispanic Stress Inventory = HSI; PTSD Symptom Checklist–Civilian Version = PCL; Multi-Sector Social Support Inventory = MSSI; Difficulty Accessing Resources Checklist = DARC; Satisfaction with Resources = SR; World Health Organization Quality of Life Scale = WHOQOL.
